# Probing Hexaminolevulinate Mediated PpIX Fluorescence in Cancer Cell Suspensions in the Presence of Chemical Adjuvants

**DOI:** 10.3390/ijms21082963

**Published:** 2020-04-22

**Authors:** Kit Man Chan, Jonathan Gleadle, Krasimir Vasilev, Melanie MacGregor

**Affiliations:** 1 Department of Engineering, University of South Australia, Adelaide, SA 5095, Australia; kit_man.chan@mymail.unisa.edu.au; 2 Department of Renal Medicine, Flinders Medical Centre, Flinders University, Bedford Park, SA 5042, Australia; jonathan.gleadle@flinders.edu.au; 3 College of Medicine and Public Health, Flinders University, Bedford Park, SA 5042, Australia; 4 Future Industries Institute, School of Engineering, University of South Australia, Adelaide, SA 5095, Australia; krasimir.vasilev@unisa.edu.au

**Keywords:** hexaminolevulinate, protoporphyrin IX, ferrochelatase, mitochondria, metabolism, 5-aminolevulinic acid (ALA), fluorescence, single cell, photodynamic, cancer

## Abstract

Exogenous administration of hexaminolevulinate (HAL) induces fluorescent protoporphyrin IX (PpIX) accumulation preferentially in cancer cells. However, the PpIX fluorescence intensities between noncancer and cancer cells are highly variable. The contrast between cancer and noncancer cells may be insufficient to reliably discriminate, especially at the single cell level in cancer diagnostics. This study examines the use of the chemical adjuvants dimethylsulphoxide (DMSO) or deferoxamine (DFO) to enhance the HAL induced PpIX accumulation in cancer cells. Our results showed that in some of the incubation conditions tested, the addition of DFO with HAL significantly increased PpIX 21 fluorescence of adherent monolayer cancer cells, but this was never the case for cells in suspension. Permeabilisation with DMSO did not increase PpIX fluorescence. Cell-to-cell interaction may well play an important role in the PpIX accumulation when suspended cells are treated in HAL and adjuvant chemicals.

## 1. Introduction

Photodynamic diagnosis (PDD) is a cancer detection technique using the selective accumulation of a fluorochrome to recognise abnormal tissue. The principle of PDD is that the exogenous administration of a photosensitiser (PS) causes malignant and/or premalignant tissues to emit a specific fluorescent signal under exposure to an appropriate light wavelength. One of the most common PS in current clinical use is 5-aminolevulinic acid (ALA) [1].

**Figure 1:**
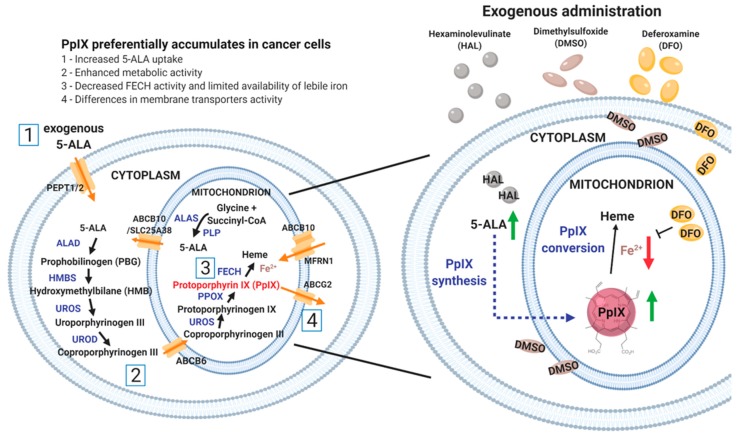
Schematic diagram of the heme biosynthesis pathway (left) and the principle of HAL mediated PpIX production enhancement with dimethylsulphoxide (DMSO) or deferoxamine mesylate salt (DFO) (right). DMSO favours the formation of pores in the cell and mitochondrion membranes, which increase the uptake of hexaminolevulinate 5-aminolevulinic acid (HAL (5-ALA)) and in turn boosts intracellular protoporphyrin IX (PpIX) synthesis (green arrow). In contrast, DFO inhibits the conversion of PpIX to heme by chelating iron (red arrow). The administration of both prodrugs attempts to enhance PpIX accumulation. The number [1 to 4] corresponds to the factors why preferentially accumulates in cancer cells when compared with normal cells.

5-ALA, a precursor of fluorescent protoporphyrin IX (PpIX), is a naturally occurring amino acid which is metabolised intracellularly to PpIX via the heme biosynthesis pathway, Figure 1. The synthesis of 5-ALA, catalyzed from succinyl-CoA and glycine by ALA synthase (ALAS), initiates the biosynthesis of heme. After several enzymatic processes, ferrous iron is chelated with PpIX by a rate-limiting enzyme, ferrochelatase (FECH), to form heme in the mitochondrion [2]. The synthetic pathway is controlled through a feedback loop mechanism which can be bypassed by adding exogenous 5-ALA. Exogenous administration of 5-ALA and its derivatives increases the accumulation of fluorescent endogenous PpIX, preferentially in tumour cells, when compared with normal cells [3,4]. When excited by blue light at 405 nm wavelength, tumour cells display red fluorescence at 620 nm because of the presence of intracellular PpIX. 5-ALA based PDD is used in the detection of a wide range of cancers, such as brain [5], ovarian [6], colorectal [7], breast [8] and bladder [9]. In urology, blue light cystoscopy PPD is now recommended by several guidelines around the world. Yet the underlying mechanisms responsible for the specificity of PpIX accumulation in tumour cells remains poorly understood [10]. To date, it has been attributed to the distinctive nature of cancer cells, primarily, to reduced FECH activity [11] and to the limited availability of iron in cancer cells [12,13] or to the reduced availability of nicotinamide adenine dinucleotide phosphate (NADPH) provision. However, the difference in PpIX fluorescence intensity between normal and cancer cells is highly variable from one cell type to another [14]. In fact, the contrast is, in some cases, insufficient to reliably discriminate between the two, especially when applying PDD principles ex vivo, at the single cell level [15,16]. To enable the development of noninvasive diagnostic methods using PDD on body fluid sediments, such as urine [17,18,19,20], practical ways to enhance the PpIX fluorescence contrast ex vivo are required [21]. Strategies to do so may well be different from those that are successful in vivo, simply because the cell microenvironments are drastically different in those two scenarios.

In this study, the 5-ALA derivative hexaminolevulinate (HAL) was used as a prodrug to increase intracellular 5-ALA concentration and boost PpIX metabolism. HAL cellular uptake through the cell membrane transporter (PEPT1/2) [22] is greater than that of 5-ALA thanks to its higher lipophilicity. As a result, it has been shown to induce intracellular PpIX accumulation in vitro at lower concentrations and with shorter incubation times than 5-ALA [23]. Membrane penetration, as a first factor determining the amount of PpIX that accumulates in cancer cells, is expected to play an important role for cells suspended in body fluids due to the increase in membrane area exposed to the prodrug for uptake. The accumulation of PpIX is subsequently dictated by a sequence of enzymatic reactions, including iron chelation [24,25]. Thus, we hypothesise that HAL-induced cancer specific fluorescence could be altered by the addition of chemical adjuvants acting either on membrane permeability or enzymatic conversion rate. HAL is approved by the Food and Drug Administration (FDA) and the European Association of Urology for bladder cancer PDD via fluorescently assisted cystoscopy, an invasive procedure. Here we aim to enhance the specificity of HAL induced PpIX concentration in malignant urothelial cells ex vivo. We studied the effect of a membrane permeant (dimethylsulphoxide (DMSO)) or an iron chelator (deferoxamine mesylate salt (DFO)) on cell monolayers and cell suspension when administered in combination with HAL (Figure 1).

## 2. Results

**Figure 2:**
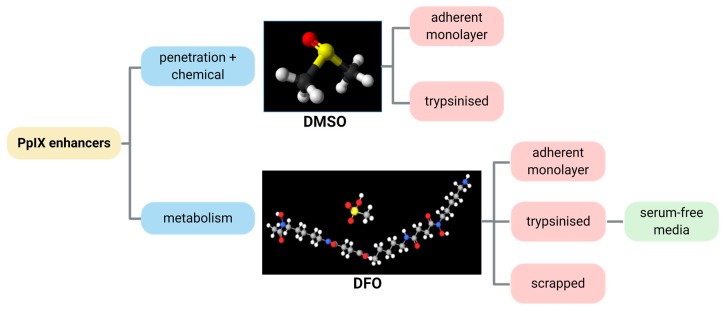
Illustrative diagram showing the process approach to improve the HAL mediated PpIX fluorescence by the chemicals DMSO and DFO.

Previous reports [26] identified the optimum ex vivo HAL incubation conditions as being 50 µM HAL for 1–2 h. We combined these incubation conditions with different DMSO or DFO treatments to identify the role of those chemical adjuvants. In this way, we intended to act on both HAL penetration rates and cell metabolic activity, as summarised in Figure 2. We tested these approaches on both adherent cell monolayers and trypsinised cells in suspension. To further improve the selectivity of PpIX accumulation towards malignant cells in suspension, we also tested cell suspensions where membrane damage was minimised (scrapped cells instead of trypsinised) and enzymatic activity boosted (using serum-free media instead of PBS).

### 2.1. DMSO

**Figure 3:**
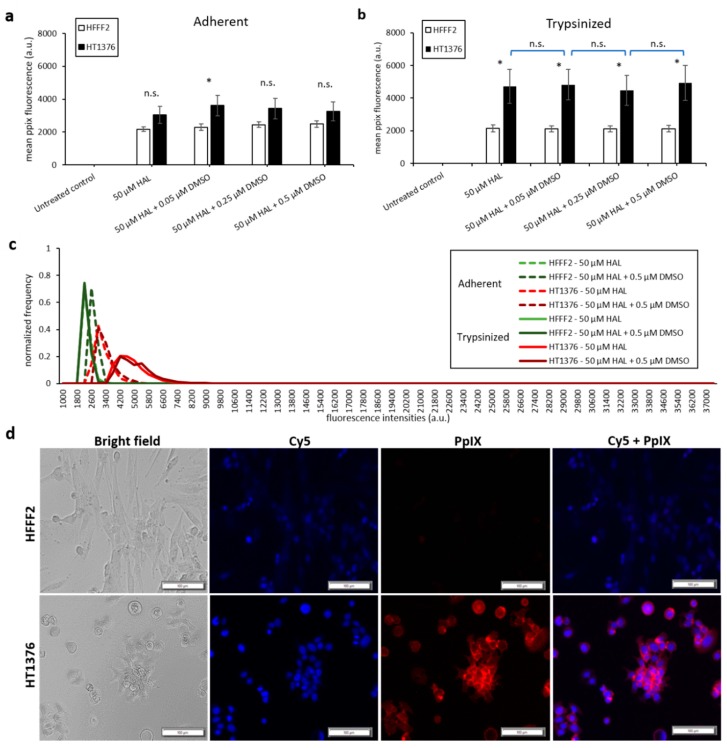
Effect of DMSO and HAL treatment on PpIX fluorescence in human bladder cancer HT1376 and human fibroblast HFFF2 cells. Cells were incubated with HAL (50 µM) alone or HAL (50 µM) and different concentrations of DMSO (0.05 to 0.5 µM) in PBS for 2 h. Mean ±SD (*n* = 3), statistical significance by ANOVA. *n.s*. = not significant and * *p* < 0.05, compared between bladder cancer HT1376 and noncancer fibroblast HFFF2 in the same conditions. PpIX fluorescence was measured in adherent (**a**) and trypsinised (**b**) cells. Results are expressed in bar and (**c**) histogram (50 µM HAL + 0.05/0.25 µM DMSO not shown) graphs. (**d**) Microscopic images showing the PpIX fluorescence in adherent monolayer bladder cancer HT1376 cells after combined treatment with HAL and 0.5 µM DMSO compared to foreskin fibroblast HFFF2 cells (trypsinised cells images not shown). Scale bars represent 100 µm, magnification is 10X.

Bladder cancer HT1376 cells displayed greater PpIX fluorescence than HFFF2, as shown in Figure 3. The difference was significant in trypsinised cells for all chemical adjuvant mix investigated (black * marker, *p* < 0.05). However, in adherent monolayer cells, the contrast in fluorescence intensity between fibroblast and bladder cancer cells was only significant (*p* < 0.05) when 0.05 µM of DMSO was added to 50 µM HAL. Nonetheless, the addition of DMSO did not significantly increase the PpIX fluorescence levels within bladder cancer HT1376 cells for any of the concentrations investigated (0.05 to 0.5 µM), regardless of the cells being in adherent monolayers (Figure 3a) or trypsinised (Figure 3b). Neither did the addition of DMSO increase the PpIX fluorescence in fibroblast HFFF2, adherent or trypsinised cells. Thus, there were no changes in the fluorescence intensity histogram following the DMSO treatment of all cells (adherent/trypsinised, HFFF2/HT1376), Figure 3c. Fluorescence microscopy images, Figure 3d, show PpIX fluorescence in adherent monolayer HT1376 cells incubated with HAL and DMSO, but not in HFFF2 cells.

**Figure 4:**
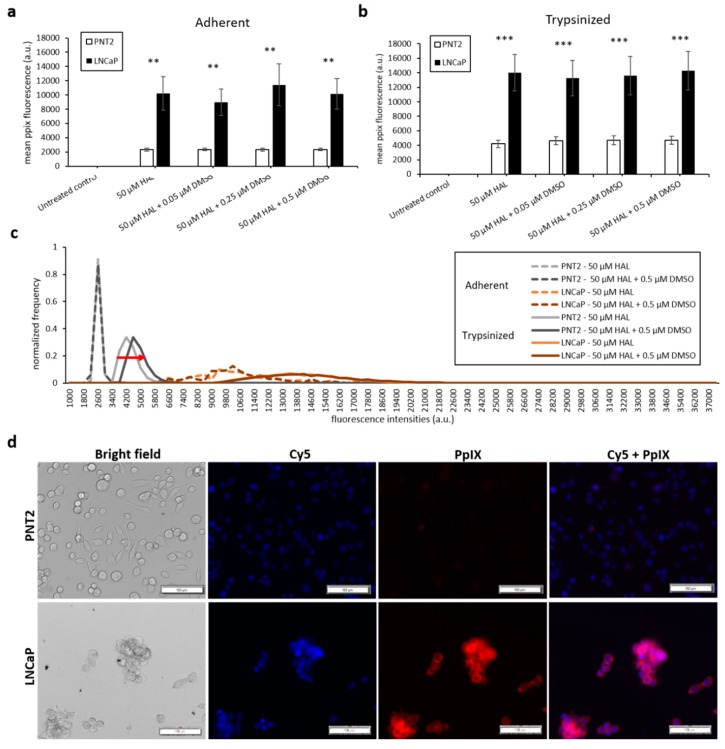
Effect of DMSO and HAL treatment on PpIX fluorescence in human prostate cancer LNCaP and human prostate PNT2 cells. Cells were incubated with HAL (50 µM) alone or HAL (50 µM) and different concentrations of DMSO (0.05 to 0.5 µM) in PBS for 2 h. Mean ± SD (*n* = 3), statistical significance by ANOVA. ** *p* < 0.01 and *** *p* < 0.001 compared between prostate cancer LNCaP and noncancer prostate PNT2 in the same conditions. PpIX fluorescence was measured in adherent and trypsinised cells. Results are expressed in (**a** and **b**) bar and (**c**) histogram (50 µM HAL + 0.05/0.25 µM DMSO not shown) graphs. (**d**) Microscopic images showing the PpIX fluorescence in adherent prostate cancer LNCaP cells after combined treatment with HAL and 0.5 µM DMSO compared to prostate PNT2 cells (trypsinised cells images not shown). Scale bars represent 100 µm, magnification is 10X.

The results of the DMSO treatment in nontumourigenic prostate PNT2 and prostate cancer LNCaP cells are shown in Figure 4. Once again, the difference in mean fluorescence intensity between normal prostatic epithelial cells and malignant cell lines was more pronounced in trypsinised cells (*p* < 0.001) than in adherent cells (*p* < 0.01), (Figure 4a,b). The addition of DMSO did not significantly increase the PpIX fluorescence of adherent monolayer PNT2 cells in any of the conditions investigated. However, in trypsinised PNT2 cells, the fluorescence intensity histogram displayed a shift toward higher PpIX intensity after the addition of 0.5 µM DMSO with HAL (Figure 4c, red arrow). This minor shift seems to indicate that the PNT2 cells were more sensitive to the presence of DMSO. The damages caused to the cell membrane may well increase the HAL uptake, and for healthy cells otherwise producing a very low level of PpIX, this led to a small increase in the fluorescence of some cells, though this was not enough to result in a statistically significant increase in the mean intensities (Figure 4a).

Thus, adding DMSO to trypsinised cells decreased the contrast between cancer and healthy cells. Using 0.25 µM DMSO with HAL produced more PpIX fluorescence in adherent LNCaP cells than other groups (Figure 4a). The resulting PpIX fluorescence histogram (Figure 4c) exhibits no apparent difference in adherent monolayer and trypsinised LNCaP cells in the parameters tested. The fluorescence images show that there was no or very little PpIX accumulated in adherent monolayer PNT2 cells, while strong PpIX fluorescence was observed in LNCaP cells as expected (Figure 4d). Overall, the addition of DMSO did not enhance the contrast between benign and malignant cell types.

### 2.2. DFO

**Figure 5:**
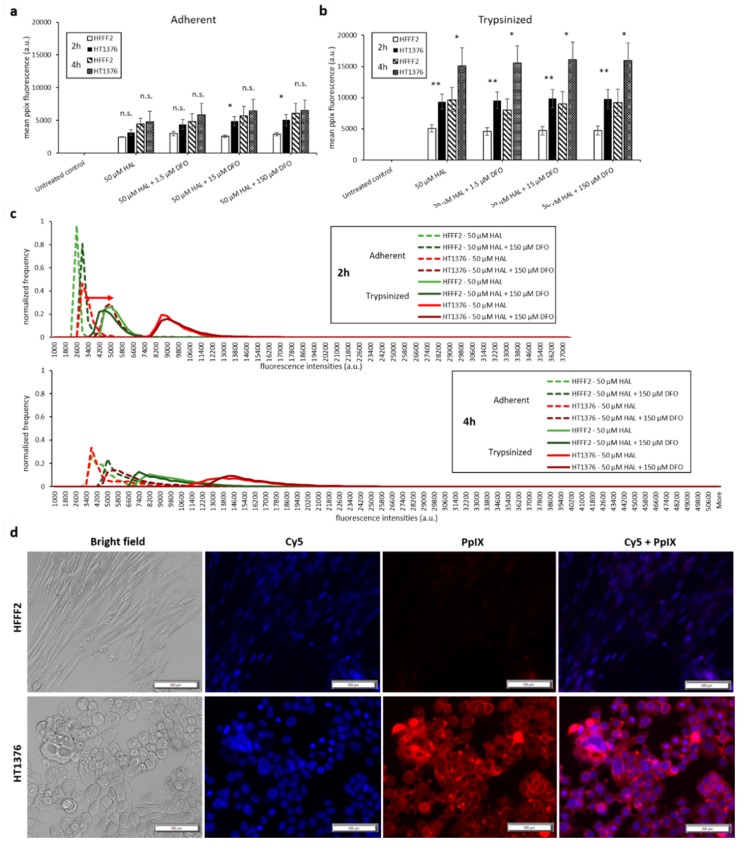
Effect of DFO and HAL treatment on PpIX fluorescence in human bladder cancer HT1376 and human fibroblast HFFF2 cells. Cells were incubated with HAL (50 µM) alone or HAL (50 µM) and different concentrations of DFO (1.5 to 150 µM) in PBS for 2 and 4 h. Mean ± SD (*n* = 3), statistical significance by ANOVA. *n.s.* = not significant, * *p* < 0.05 and ** *p* < 0.01, compared between bladder cancer HT1376 and human fibroblast HFFF2 cells in the same conditions. PpIX fluorescence was measured in adherent and trypsinised cells. Results are expressed in (**a** and **b**) bar and (**c**) histogram (50 µM HAL + 1.5/15 µM DFO not shown) graphs. (**d**) Microscopic images showing the PpIX fluorescence in adherent bladder cancer HT1376 cells after combined treatment with HAL and 150 µM DFO compared to fibroblast HFFF2 cells (trypsinised cells images not shown). Scale bars represent 100 µm, magnification is 10X.

The same experimental procedure was undertaken to evaluate the effect of DFO on HAL induced PpIX fluorescence. DFO was prepared at concentrations ranging between 1.5 and 150 µM and incubated with the cells for 2 and 4 h. DFO treatment led to a greater increase in PpIX fluorescence of adherent monolayer bladder cancer cell line HT1376 than on adherent fibroblast HFFF2 cells for both 2 and 4 h time points, leading to a statistical difference between cancer and healthy cell for 2 h incubation with 15 and 150 µM DFO (Figure 5a). Again, the overall intensity of the fluorescence emitted from all cell types was much greater for trypsinised than adherent monolayer cells (Figure 5b). However, the PpIX fluorescence levels remained unchanged for both trypsinised HFFF2 and HT1376 cells when combining HAL with DFO treatment (Figure 5b). Histograms showed distinctly resolved PpIX fluorescence intensity peaks for trypsinised fibroblast HFFF2 and bladder cancer HT1376 cells after HAL incubation, in good agreement with our previous report [26]. The effect of DFO was clearly observed through a shift in the peak maximum when adherent HT1376 cells were co-incubated with and without DFO plus HAL in 2 h rather than in HFFF2 cells (Figure 5c, red arrow).

**Figure 6:**
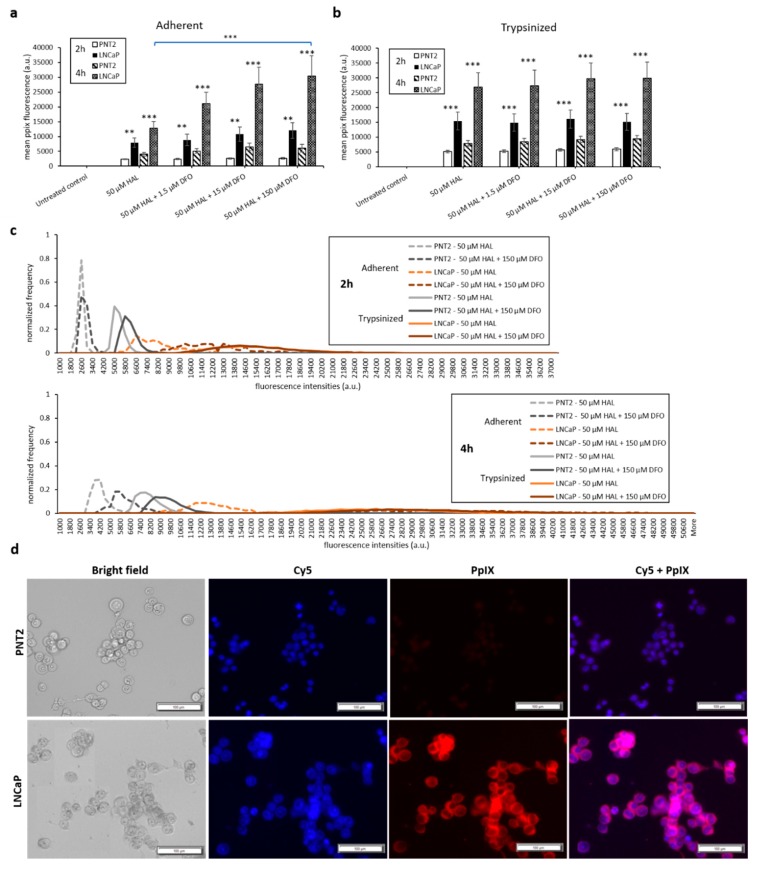
Effect of DFO and HAL treatment on PpIX fluorescence in human prostate cancer LNCaP and human prostate PNT2 cells. Cells were incubated with HAL (50 µM) alone or HAL (50 µM) and different concentrations of DFO (1.5 to 150 µM) in PBS for 2 and 4 h. Mean ± SD (*n* = 3), statistical significance by ANOVA. ** *p* < 0.01 and *** *p* < 0.001 compared between prostate cancer LNCaP and prostate PNT2 cells in the same conditions. PpIX fluorescence was measured in adherent and trypsinised cells. Results are expressed in (**a** and **b**) bar and (**c**) histogram (50 µM HAL + 1.5/15 µM DFO not shown) graphs. (**d**) Microscopic images showing the PpIX fluorescence in adherent prostate cancer LNCaP cells after combined treatment with HAL and 150 µM DFO compared to prostate PNT2 cells (trypsinised cells images not shown). Scale bars represent 100 µm, magnification is 10X.

The addition of DFO also led to a significant increase in PpIX fluorescence for prostate cancer cell lines. Specifically, HAL plus DFO treatment for 4 h resulted in a substantial enhancement of PpIX fluorescence in adherent monolayer prostate cancer LNCaP cells (*p* < 0.001). However, for adherent monolayer prostate PNT2 or trypsinised LNCaP and PNT2 cells, the addition of DFO did not enhance PpIX fluorescence significantly for 2 and 4 h (Figure 6a,b).

DFO enhances HAL-induced PpIX fluorescence in both bladder cancer HT1376 and prostate cancer LNCaP cell monolayer, especially in LNCaPs but not in fibroblast HFFF2 and prostate PNT2 cells. The addition of increasing amounts of DFO (15 and 150 µM) caused a significant difference (*p* < 0.05) in mean PpIX fluorescence intensity between adherent monolayer bladder cancer HT1376 and fibroblast HFFF2 cells at the 2 h time point. Similarly, the PpIX fluorescence difference between adherent monolayer prostate cancer LNCaP and prostate PNT2 cells was greater in the presence of DFO, at all doses investigated, in both 2 (*p* < 0.01) and 4 (*p* < 0.001) hour time points.

Changes in HAL-induced PpIX fluorescence following the addition of DFO appear to depend not only on cell type but also on the microenvironment. DFO treatment enhanced the difference between malignant and benign cells in monolayers, but it did not change the PpIX fluorescence in any of the trypsinised cells in suspension. Used in these conditions, the usefulness of DFO would, therefore, be limited for a liquid biopsy of cells in suspension. A key difference between trypsinised cells and those found in monolayers is the degree of cell integrity and stress. The viability and metabolic activity of trypsinised cells may be compromised by both the chemical dissolution of membrane proteins by trypsin and also extended exposure to PBS media.

### 2.3. Serum-Free Media and Scraping

**Figure 7:**
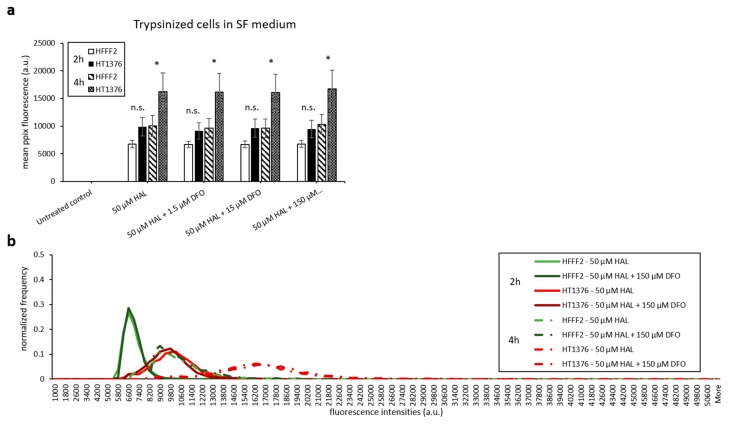
Effect of DFO and HAL treatment on PpIX fluorescence in human bladder cancer HT1376 and human fibroblast HFFF2 cells. Trypsinised cells were incubated with HAL (50 µM) alone or HAL (50 µM) and different concentrations of DFO (1.5 to 150 µM) in their respective serum-free (SF) medium for 2 and 4 h. Mean ± SD (*n* = 3), statistical significance by ANOVA. *n.s.* = not significant and * *p* < 0.05, compared between bladder cancer HT1376 and fibroblast HFFF2 in the same conditions. PpIX fluorescence was measured, and results are expressed in (**a**) bar and (**b**) histogram graphs.

**Figure 8:**
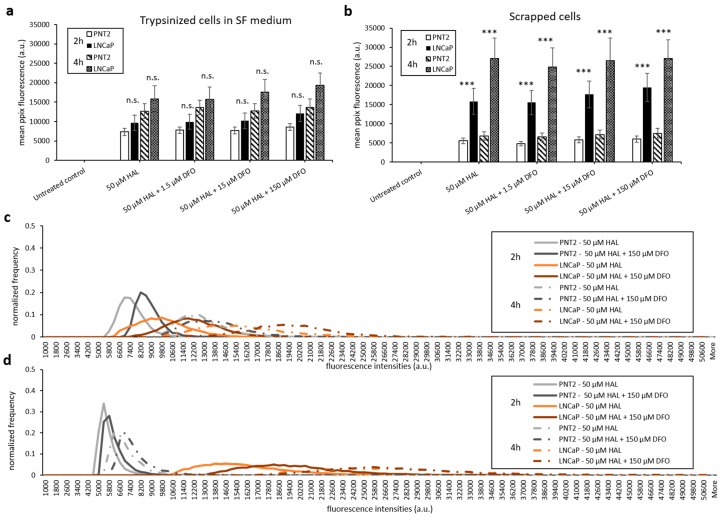
Effect of DFO and HAL treatment on PpIX fluorescence in human prostate cancer LNCaP and human prostate PNT2 cells. Trypsinised cells were incubated with HAL (50 µM) alone or HAL (50 µM) and different concentrations of DFO (1.5 to 150 µM) in their respective serum-free (SF) medium for 2 and 4 h (**a** and **c**). The DFO and HAL incubation condition for scrapped cells was the same as trypsinised cells but kept in PBS instead of SF medium (**b** and **d**). Mean ± SD (*n* = 3), statistical significance by ANOVA. *n.s*. = not significant and *** *p* < 0.001 compared between prostate cancer LNCaP and prostate PNT2 cells in the same conditions.

With the aim of maintaining cell metabolism and viability, we further investigated the effect of DFO on either cells harvested by scraping or trypsinised cells kept in serum-free medium. As shown in Figure 7 and Figure 8, there were no significant changes in HAL induced PpIX fluorescence under all DFO incubation conditions investigated. In addition, just like trypsinised cells in PBS, increased PpIX fluorescence was observed over time in all cell lines, as expected. This agrees with our previous findings that longer incubation time leads to increased PpIX accumulation. PpIX accumulation with time was more pronounced in malignant (HT1376/LNCaP) cells compared to the benign (HFF/PNT2) cells. However, in fibroblast HFFF2 and prostate PNT2 cells, the PpIX fluorescence intensities were much higher when cells incubated in serum-free medium than in PBS. It is worth noting, however, that the untreated control cells did not display any background fluorescence in the presence of media. It is, therefore, unlikely that autofluorescence from the media components is responsible for this increase. The mean PpIX fluorescence increased by 30–35% in 2 h incubation with HAL and DFO and by 20–40% at the 4 h time point in both noncancer trypsinised cells when cells were kept in serum-free medium compared to PBS. In contrast, prostate cancer LNCaP cells display a decreased PpIX fluorescence by 50–60% in serum-free medium for both time points. A faint increase in PpIX fluorescence was also observed in bladder cancer HT1376. Overall, the contrast between healthy and malignant cells was, therefore, reduced in serum-free media, becoming nonsignificant for all conditions but 4 h for bladder cancer cells (*p* < 0.05).

Only prostate cells were harvested by scraping for testing. Comparing trypsinised (Figure 6b) and scrapped cells in PBS (Figure 8b), we found that both cell lines had a similar level of HAL induced PpIX fluorescence. The method of harvesting cells did not change the PpIX fluorescence at both 2 and 4 h time points.

A summarised result in all cell types and conditions is presented in Table 1.

## 3. Discussion

It is well-known that PpIX accumulates more in malignant tissue than in benign tissue following exogenous administration of 5-ALA and its derivatives [27]. The same phenomenon has also been demonstrated in cell cultures, where cells are grown in monolayers [12]. In previous work, however, we showed that this effect is more complex at the single cell level. Thus, while we identified suitable conditions to discriminate between malignant and benign cells in suspension, we also found that the HAL induced fluorescence was inconsistent from cells to cells and in different cell lines. In particular, we found that the adjuvant effect of Nuclear Red™, a DNA selective dye solvated in DMSO, enhanced PpIX fluorescence by 4.5 times in bladder cancer cells compared to noncancer fibroblast cells [26]. The Cy5 (nuclear red) fluorescence appears to be stronger and more diffuse in noncancer fibroblasts than in human bladder cancer cells; this phenomenon has also been observed between nontumourigenic prostate PNT2 and prostate cancer LNCaP cells. This could be the result of photodamages caused by PpIX to the internal cells’ organs, allowing the nuclear stain to spread outside the nuclei. While this observation warrants further investigation, it goes beyond the scope of the present study. Other groups have shown that DMSO enhances the permeability of cell membranes for drug delivery [28,29]. Another reported property of DMSO is to act as a cell differentiation potentiator. Results from an in vitro experiment indeed suggested that DMSO increases the heme synthase activities in murine erythroleukemia cells, leading to the accumulation of coproporphyrin III and PpIX [30]. For these reasons, we hypothesised that a DMSO supplement could lead to the specific enhancement of HAL mediated PpIX fluorescence in cancer cells compared to healthy cells.

Yet, our results showed no enhancement of PpIX fluorescence on any of the adherent and trypsinised cell lines treated with a combination of HAL and DMSO. One possible explanation for this is that, due to its membrane-permeant properties, DMSO does not only increase the uptake of exogenous 5-ALA/HAL but also enhances the excretion of PpIX from the cells. An effect that could be enhanced when cells are in suspension compared to monolayers and one that could be equally impacting healthy and cancer cells. In fact, the presence of DMSO seems to neutralise or even counteract the preferential accumulation of PpIX in malignant cells in suspension. We thus tested a different chemical approach which would not compromise the lipid bilayer integrity, DFO.

DFO is an iron chelator. It has been utilised to remove labile iron and, therefore, inhibit the conversion of PpIX to heme in human adenocarcinoma cells in vitro [31]. As such, its mode of action is to decrease the conversion of intracellular PpIX to heme by specifically limiting the activity of FECH. Our results show that treatment with DFO did enhance PpIX fluorescence on adherent monolayer prostate cancer LNCaP cells but not in trypsinised cells. These findings indicate that the effect of DFO was absent on cells in suspension, possibly because the cells were stressed by prolonged exposure to PBS and/or because the cell membrane integrity had been compromised by trypsinisation. To untangle these two potential pitfalls, we explored two independent ways of generating cell suspensions where 1) cell metabolic activity was maintained by using media instead of PBS, and 2) membrane integrity was preserved by gently scraping the cells to resuspend them instead of using trypsin.

Serum-free media, as a cell growth supplement, improves the overall intracellular metabolic activity. Interestingly though, the mean PpIX fluorescence increased only for the noncancer cells (HFFF2 and PNT2) when incubated in serum-free media compared to PBS, but it did not for bladder cancer HT1376, and even decreased for prostate cancer LNCaPs. In this experiment, all cells had been trypsinised, and so all cell membranes were compromised in such a way that both healthy and cancer cells could largely and equitably uptake the HAL. Our results, therefore, indicate that acting on metabolic activity had an effect, but since healthy cell lines were more responsive to the serum-free media boost than cancer cell lines, this effect was detrimental to increasing the contrast in PpIX fluorescence.

We then used scraping to resuspend cells to avoid denaturation of the cell membrane. Scrapped cells displayed fluorescence of intensity comparable to that of trypsinised cells, around 27,000 a.u., which is approximately 50% more than adherent cells exposed to the corresponding HAL treatment. A significant increase in PpIX fluorescence occurs when the whole cell surface area is freely exposed to exogenous HAL in suspension, regardless of whether the cell membrane has been damaged by trypsin or not. What is more, the addition of DFO did not increase the PpIX fluorescence of scrapped cells any further, as it did for adherent monolayer cells (e.g., DFO treatment increased PpIX fluorescence of adherent LNCaP cells from 13,000 a.u. in 50 µM HAL to 21,000 a.u. in 50 µM HAL + 1.5 µM DFO at 4 h). In other words, the intensity of PpIX fluorescence occurring when cells are in suspension is so large that no further enhancement can be achieved by adding DFO, possibly because the PpIX level, FECH activity or both are already at saturation.

Demonstrating that DFO has no effect on trypsinised or scrapped cells in suspension has important implications for the development of PDD in ex vivo diagnostic, as it implies that strategies that may well work in tissues and cultured cell monolayers may not apply in liquid biopsy conditions. In vivo, DFO-mediated iron chelation has increased 5-ALA-induced PpIX fluorescence for U251 glioma [32] and oral squamous cell carcinoma [33]. In optimum cell culture conditions, it did too for normal skin fibroblasts, keratinocytes HaCaT and larynx carcinoma Hep-2 [24]; colon adenocarcinoma WiDr and erythroleukemia K562 [34]. Our results show that DFO enhances PpIX fluorescence in cell monolayers, but it does not when the same cells of adherent monolayer nature are forced into suspension. Different mechanisms, such as uptake rates, enzymatic activities or cell-to-cell interaction, have been identified as playing a role in the 5-ALA-induced PpIX photoactivation following exogenous DFO administration [24,35,36]. Yet, our attempt at enhancing the metabolic activities and maintaining membrane integrity did not increase the PPIX fluorescence of suspended cells any further. The cell-to-cell interaction, which is absent for cells in suspension, could be key for the DFO+HAL treatment to raise the PpIX signal. Therefore, to enhance the contrast between healthy and cancer cells in liquid biopsy, different strategies will be needed. Focusing on decreasing the PpIX fluorescence of healthy cells rather than increasing that of cancerous cells, could be more appropriate because cancer cells may already be at PpIX saturation in suspension. This could be achieved by acting on PpIX transporter inhibitors to modify the PpIX efflux or using adjuvants, which regulates other enzymes involved in PpIX synthesis [10,21].

## 4. Materials and Methods

### 4.1. Reagents

Hexaminolevulinate (HAL) hydrochloride, dimethylsulfoxide (DMSO), deferoxamine mesylate salt (DFO) and phosphate-buffered saline (PBS) tablets were purchased from Sigma-Aldrich (Castle Hill, Australia). Nuclear red™ LCS1 (Cat# 17542) was obtained from AAT Bioquest^®^ (Sunnyvale, CA 94085, USA).

### 4.2. Cell Lines

Three human bladder carcinoma cell lines HT1376 (cat no.87032402), HT1197 (cat no.87032403) and RT4 (cat no.91091914), human prostate carcinoma cell line LNCaP clone FGC (cat no.89110211), human normal prostate cell line PNT2 (cat no.95012613), human foetal foreskin normal fibroblast cell line HFFF2 (cat no.86031405), were all supplied by the European Collection of Cell Cultures (ECACC; Salisbury, United Kingdom) and purchased from CellBank Australia (Westmead, NSW, Australia). PNT2 and HFFF2 as normal cell lines were meant to be noncancer. Cells were cultured in MEME + 1% MEM nonessential amino acid solution (HT1376 and HT1197), McCoy’s 5A (RT4) and RPMI-1640 (LNCaP and PNT2), culture medium were obtained from Sigma-Aldrich (Castle Hill, Australia). HFF cells were cultured in DMEM from Thermo Fisher Scientific (Scoresby, Australia). All culture growth medium were supplemented with 10% fetal calf serum and 1% (*v/v*) penicillin/streptomycin (Thermo Fisher Scientific, Scoresby, Australia). Cells were cultured at 37 °C in a humidified atmosphere containing 5% CO_2_.

### 4.3. HAL Based Treatment with DMSO

*Adherent monolayer cells*. All cells were seeded in 96-well plates with their respective culture medium at 37 °C for 24 h. After 24 h, the cells were incubated with 50 µM HAL and different volumes (0 to 10 µL) of 1:100 diluted DMSO in PBS at 37 °C in the dark for 2 h. The volume of DMSO added was determined from our previous study [26].

*Trypsinised cells in suspension*. Cells were trypsinised by 1X trypsin-EDTA for 5 to 10 min. After trypsinisation, serum-containing medium was added to the trypsinised cell suspension to stop further tryptic activity. Then the trypsinised cell suspension was centrifuged in 1500 rpm for 5 min at room temperature. The supernatant was removed, and the cell pellet was resuspended in PBS. Sample incubation was performed as described above in Adherent monolayer cells. After incubation, the cells were resuspended, and 100 µL per well was aliquoted into 96-well plates.

### 4.4. HAL Based Treatment with DFO

*Adherent monolayer cells*. All cells were seeded in 96-well plates and cultured at 37 °C for 24 h. After 24 h, cells were incubated with 50 µM HAL and 0 to 150 µM DFO in PBS at 37 °C in the dark for 2 or 4 h.

*Trypsinised cells in suspension*. Cells were trypsinised and resuspended, as we mentioned in Section 4.3 Trypsinised cells in suspension above. Then, the cells were incubated with 50 µM HAL and 0 to 150 µM DFO in PBS or their respective serum-free medium at 37 °C in the dark for 2 or 4 h. After incubation, the cells were resuspended, and 100 µL per well was aliquoted into 96-well plates.

*Scrapped cells in suspension*. Cells were gently detached from culturing flask using a cell scraper into the medium. The sample incubation with DFO was then carried out as described above in Trypinised cells in suspension.

### 4.5. Nuclear Red

*Both Adherent monolayer cells and trypinised cells in suspension.* After the HAL based treatment with either DMSO or DFO, cells were stained with 0.5 µM nuclear red for 15 min. Fluorescence was excited at 622 nm and emitted at 645 nm with a Cy5 optical channel for cellular imaging. Cy5 and PpIX imaging were performed simultaneously under the custom made IX83 inverted fluorescent microscope (Olympus, Japan). Nuclear red is here added for the purpose of localizing cells that may not display HAL-induced PpIX fluorescence.

### 4.6. PpIX Fluorescence Measurement

Images of PpIX fluorescence were obtained using a custom made IX83 inverted fluorescent microscope (Olympus, Japan) equipped with a specially designed filter cube (Chroma). PpIX fluorescence was excited at 405 nm and emitted at 600 nm using appropriate LED lamps and a custom PpIX optical channel long pass filters. The fluorescence intensity was measured using Image-Pro Premier software (Media Cybernetics Inc., Rockville, MD 20852, USA). For cellular fluorescence measurement, background noise was removed by intensity threshold adjustment in image pro software. Using consistent detector system settings, objects of interest (PpIX fluorescent cells) that were distinguishable from the background were counted. The average intensity of each object was calculated over all pixels contained within the object contour, using a built-in software algorithm. The mean and standard deviation values were calculated by one-way ANOVA and standard variations test for each condition and each cell line (Minitab 18). The average fluorescence intensities of each cell in triplicate wells (*n* = 3) were recorded and were used to construct a normalised histogram. The histogram represents the average statistical distribution of cell fluorescence intensity data in each sample.

## 5. Conclusions

HAL-assisted cystoscopy is a recent breakthrough in urology, but it remains an invasive diagnostic method. Using the same photodynamic principle, cancer cells shed in urine could be identified in a noninvasive manner if the contrast between healthy and cancer was marked enough at the single cell level. Here, we tested a chemical adjuvant acting on cell permeability and enzymatic activity with the aim to enhance HAL-induced PpIX fluorescence in urothelial cancer cells. The differences in PpIX fluorescence were investigated prior to and following the exogenous administration of HAL with DMSO/DFO on adherent cells in monolayers and in suspension. Bladder and prostate cancer cells were tested as well as healthy prostate epithelial cells and fibroblats. The cancer cells consistently displayed higher fluorescence intensity than healthy cells. When combined with HAL, DFO adjuvant only increased the PpIX fluorescence on cancer cells in monolayers but not on cells in suspension. Permeabilisation with DMSO did not increase PpIX fluorescence. Neither DMSO nor DFO is suitable to enhance the contrast in PpIX fluorescence when cancer and normal cells are in suspension. The results indicate that the cell microenvironment may well play a more important role than either difference in HAL uptake or enzymatic activity in the specificity of PpIX accumulation in cancer cells.

## Figures and Tables

**Table:** (**A**)

Condition	Cell Line	50 µM HAL (Alone)	50 µM HAL + 0.05 µM DMSO	50 µM HAL + 0.25 µM DMSO	50 µM HAL + 0.5 µM DMSO
Adherent monolayer	HFFF2	–	–	–	–
	HT1376	–	↑	–	–
	PNT2	–	–	–	–
	LNCaP	–	↓	↑	–
Trypsinised cells in PBS	HFFF2	–	–	–	–
	HT1376	–	–	–	–
	PNT2	–	–	–	–
	LNCaP	–	↓	–	–

**Table:** (**B**)

		2 h				4 h			
Condition	Cell Line	50 µM HAL (Alone)	50 µM HAL + 1.5 µM DFO	50 µM HAL + 15 µM DFO	50 µM HAL + 150 µM DFO	50 µM HAL (Alone)	50 µM HAL + 1.5 µM DFO	50 µM HAL + 15 µM DFO	50 µM HAL + 150 µM DFO
Adherent monolayer	HFFF2	–	↑	–	↓	–	–	↑	↑
	HT1376	–	↑	↑	↑	–	↑	↑	↑
	PNT2	–	–	–	–	–	↑	↑	↑
	LNCaP	–	↑	↑	↑	–	↑	↑	↑
Trypsinised cells in PBS	HFFF2	–	↓	–	–	–	↓	↓	–
	HT1376	–	–	–	–	–	–	↑	↑
	PNT2	–	–	↑	↑	–	↑	↑	↑
	LNCaP	–	–	↑	–	–	–	↑	↑
Tryspinised cells in serum-free media	PNT2	–	–	–	↑	–	↑	–	↑
	LNCaP	–	–	↑	↑	–	–	↑	↑
Scrapped	PNT2	–	↓	–	–	–	–	–	↑
	LNCaP	–	–	↑	↑	–	↓	↓	–

The intensity value measured at 50 µM HAL (alone) in each cell line and each condition was set as the baseline, if value measured ≥ baseline + 500 was considered as increase (↑); otherwise if value measured ≤ baseline – 500 was considered as decrease (↓). When value is within baseline ± 499 was considered as no change (–).
